# Nrf2/HO‐1, NF‐κB and PI3K/Akt signalling pathways decipher the therapeutic mechanism of pitavastatin in early phase liver fibrosis in rats

**DOI:** 10.1111/jcmm.18116

**Published:** 2024-01-12

**Authors:** Marawan A. Elbaset, Bassim M. S. A. Mohamed, Alyaa Hessin, Sahar S. Abd El‐Rahman, Tuba Esatbeyoglu, Sherif M. Afifi, Hany M. Fayed

**Affiliations:** ^1^ Department of Pharmacology Medical Research and Clinical Studies Institute, National Research Centre Giza Egypt; ^2^ Department of Pathology, Faculty of Veterinary Medicine Cairo University Giza Egypt; ^3^ Department of Molecular Food Chemistry and Food Development, Institute of Food Science and Human Nutrition Gottfried Wilhelm Leibniz University Hannover Hannover Germany; ^4^ Pharmacognosy Department, Faculty of Pharmacy University of Sadat City Sadat City Egypt

**Keywords:** fibrosis, NF‐κB, oxidative stress, PI3k, Nrf2, signalling pathway, statin

## Abstract

Liver fibrosis is a common chronic hepatic disease. This study aimed to investigate the effect of pitavastatin (Pit) against thioacetamide (TAA)‐induced liver fibrosis. Rats were divided into four groups: (1) control group; (2) TAA group (100 mg/kg, i.p.) three times weekly for 2 weeks; (3 and 4) TAA/Pit‐treated group, in which Pit was administered orally (0.4 and 0.8 mg/kg/day) for 2 weeks following TAA injections. TAA caused liver damage manifested by elevated serum transaminases, reduced albumin and histological alterations. Hepatic malondialdehyde (MDA) was increased, and glutathione (GSH) and superoxide dismutase (SOD) were decreased in TAA‐administered rats. TAA upregulated the inflammatory markers NF‐κB, NF‐κB p65, TNF‐α and IL‐6. Treatment with Pit ameliorated serum transaminases, elevated serum albumin and prevented histopathological changes in TAA‐intoxicated rats. Pit suppressed MDA, NF‐κB, NF‐κB p65, the inflammatory cytokines and PI3K mRNA in TAA‐intoxicated rats. In addition, Pit enhanced hepatic antioxidants and boosted the nuclear factor erythroid 2–related factor 2 (Nrf2) and heme oxygenase‐1 (HO‐1) mRNA. Moreover, immunohistological studies supported the ability of Pit to reduce liver fibrosis via suppressing *p*‐AKT expression. In conclusion, Pit effectively prevents TAA‐induced liver fibrosis by attenuating oxidative stress and the inflammatory response. The hepatoprotective efficacy of Pit was associated with the upregulation of Nrf2/HO‐1 and downregulation of NF‐κB and PI3K/Akt signalling pathways.

## 1 INTRODUCTION

Liver fibrosis is linked to severe morbidity and mortality around the globe.^1^, ^2^ A worldwide prevalence of 1.5 billion cases is estimated and accounts for more than a million deaths annually.^3^, ^4^ Viral or parasite infections are the leading causes of liver fibrosis in developing nations, whereas excessive alcohol intake is the primary factor in industrialized nations.^5^, ^6^ Autoimmune and metabolic diseases, drug‐associated disorders and hereditary conditions are additional aetiologies of hepatic fibrosis.^7^, ^8^, ^9^, ^10^ Regardless of their diverse causes, such injuries drive inflammation, and liver fibrosis becomes inevitable sequelae of all chronic liver diseases.^11^, ^12^

Reiterated liver insults lead to cellular damage, activation of myofibroblasts and persistent inflammation.^13^ As a result, the wound healing response becomes abnormal, and an increased amount of reactive oxygen species (ROS) is produced, which in turn, triggers the massive generation of inflammatory mediators, such as chemokines, cytokines, and multiple growth factors.^14^ Increased numbers of immune and inflammatory cells may be attracted by chemokines; once there, cytokines and growth factors can bind to their specific receptor, stimulating the production of several transcription factors and proteins.^15^ Consequently, regulatory pathways that support normal liver functions, cell development, proliferation and differentiation will also be disrupted. Collagen, elastin, and glycoproteins, among other extracellular matrix components (ECMs), will be made in large quantities, deposited and regenerated in the peri‐sinusoidal area.^14^ The aberrant overexpression of the enzymatic breakdown ‘matrix metalloproteinases (MMPs)’ and their corresponding antagonists causes matrix remodelling to begin. Moreover, the expression of genes that govern the production of interleukins, enzymes and growth factors is constantly upregulated.^15^ This causes a vicious cycle of liver damage and repair that finally results in the disruption of liver processes, chronic inflammation and, ultimately, liver fibrosis.^3^, ^16^, ^17^

If untreated, fibrosis can lead to cirrhosis, hepatic failure and hepatocarcinoma and potentially cause mortality. This process typically takes decades (about 20–30 years), although it can advance quickly, as in the case of biliary atresia, drug‐induced liver damage, HIV/HCV coinfection, or HCV infections following liver transplantation.^18^ A great deal of work has been done to understand the pathophysiology of fibrosis, which has led to the identification of prospective targets for antifibrotic drugs that could either slow down or reverse fibrosis.^19^

Pitavastatin is a cholesterol‐lowering agent (statin) that was approved in 2009. It is also one of the foundations for treating and preventing atherosclerotic cardiovascular disease.^20^ Similar to other members of its class (statins), Pit works by inhibiting 3‐hydroxy‐3‐methylglutaryl coenzyme A (HMG‐CoA) reductase in a competitive manner; it prevents HMG‐CoA from being converted to mevalonic acid, the cholesterol precursor.^21^ More than 200 million users around the globe receive statins and have experienced positive effects, including lowering cardiovascular events and mortality.^22^ A great deal of recent evidence has shown that statin administration in patients with preexisting chronic liver conditions such as fibrosis, cirrhosis and hepatocellular carcinoma exerts no harmful effect on the liver.^23^ In fact, clinical trials on patients with chronic liver disease proved that liver enzymes were lower in the statin‐treated group.^22^, ^23^, ^24^ Some studies postulated that the beneficial action of statins, known as pleiotropic effects, would return to the inhibitory effect on cell proliferation, the anti‐inflammatory action or the improvement in endothelial function, and the vaso‐protective effect.^25^, ^26^ Herein, we conducted a study to decipher the cell signalling pathway underlying the therapeutic effect of Pit in liver fibrosis; and to delineate a specific mechanism of action to statins in chronic liver diseases.

## 2 MATERIALS AND METHODS

### 2.1 Animals

Wistar rats weighing 150–200 g and 5 months old were acquired from the Animal House Colony at the National Research Centre (NRC, Egypt). Twenty‐four rats were housed on a 12‐h light/dark cycle at ambient temperature (25°C). The animals were treated in accordance with national and international ethical standards. All the experiments followed the ethical guidelines established by the NRC's Committee on Animal Care and Use's ethics committees (Reg. No. 1041112022).

### 2.2 Chemicals

For induction of thioacetamide (TAA) toxicity, TAA was bought from Sigma‐Aldrich (St Louis, MO, USA). For Pit treatments, Pit was acquired from Western pharmaceutical industries, Egypt. Every other chemical used in the experiments had the highest purity and analytical grade. Freshly suspended Pit was orally administered at 0.4 and 0.8 mg/kg in a 1% Tween 80 solution.

### 2.3 Experimental design

The rodents were split into four groups, each containing six animals; one was assigned as the negative control, one positive control, in addition to two Pit treatment groups. Rats in Group 1 (negative control group) were given an intraperitoneal (IP) injection of saline three times a week for two consecutive weeks. Rats in Group 2 (TAA group) received an intraperitoneal (IP) injection of TAA (100 mg/kg) three times a week for two consecutive weeks to cause liver fibrosis.^27^ Rats in groups three and four were given Pit orally ‘0.4 and 0.8 mg/kg’^28^ every day for 2 weeks following the TAA injection.

### 2.4 Preparation of serum samples

The serum sampling was performed as per Metwaly et al.; the rats were deprived of food overnight. Subsequently, blood samples were taken, and the serum was separated by spinning the samples in a centrifuge at 3000 rpm and 4°C for 5 min. The resulting serum was then stored at −80°C for future use in the analysis of biochemical parameters.^29^

### 2.5 Liver tissue collection

Liver tissues were gathered and rinsed with a cold saline solution. They were divided into three portions. The initial portion was immediately frozen in liquid nitrogen and stored at −80°C for extracting mRNA. The second portion was homogenized with a mixture of 100 mg of tissue and 1 mL of iced 0.5% potassium chloride. This was followed by 1 min sonication and 10 min centrifugation at 3000 rpm at 4°C. Subsequently, the resulting supernatant was separated and preserved at −80°C. This portion was intended for assessing hepatic glutathione (GSH) and MDA levels using colorimetric kits, as well as for measuring NFκB, p‐NFκB, TNF‐α, IL‐6, Nrf2, and HO‐1 levels using enzyme‐linked immunosorbent assay (ELISA). The last portion was fixed in 10% buffered formalin, which was necessary for conducting further histopathological and immune‐histochemical examinations.

### 2.6 Liver function tests

To evaluate liver function, serum ALT, AST, and albumin levels were measured colorimetrically using commercial kits (Bio‐diagnostic® kits Cat# AS 10 61 and AL 10 31, Cairo, Egypt).

### 2.7 Liver oxidative stress markers

The measurement of GSH content and MDA level ‘Catalog# K464‐100 and K739‐100, BioVision, Milpitas Boulevard, Milpitas, USA’, was performed calorimetrically according to the manufacturer's instructions.

### 2.8 ELISA assay

Protein concentrations were measured using commercially available enzyme‐linked immunosorbent assay (ELISA) kits; NFκB, p‐NFκB (Catalogue# MBS453975, MyBioSource, Inc., San Diego, CA 92195‐3308, USA), TNF‐α (Catalogue# SL0889Mo, Sunlong Biotec Co. LTD, Zhejiang, China), IL‐6 (Catalogue# K739‐100, BioVision, Milpitas Boulevard, Milpitas, USA), Nrf2 (Catalogue# EH3417, Wuhan Fine Biotech Co., Ltd, China (430206)), and HO‐1 (Catalogue# E4525‐100, 55 S. Milpitas Blvd., Milpitas, CA 95035 USA). The manufacturer's instructions were followed for each ELISA kit.

### 2.9 Histopathological and immune‐histochemical examinations

According to the procedure of El‐Said et al., the tissue was embedded in 10% buffered formalin, liver tissues were fixed for 24 h. Then, dehydrated in different grades of alcohol, cleared in xylene, and embedded in paraffin wax. Using hematoxylin and eosin stain, the paraffin sections (4 μm) were stained. To avoid bias, the cells were examined by a blinded pathologist using a light microscope.^30^

### 2.10 Immune‐histochemical examination of *p*‐AKT expression

The other paraffin section from each group was used for immunohistochemical detection of the expression of *p*‐AKT in various experimental groups using avidin‐biotin‐peroxidase according to the method described by.^31^

For the purpose of identifying a bound antigen and antibody, liver cuts were treated with antibodies for *p*‐AKT (Abcam, Cambridge, MA, USA) at a dilution of 1:200 (*v/v*) and (Vactastain ABC peroxidase kit, Vector Laboratories, Burlingame, USA). Chromagen 3, 3‐diaminobenzidine tetrahydrochloride was used to visualize each marker's expression (DAB, St Louis, MO, USA).

### 2.11 Statistical analysis

Before the statistical analysis, data values were checked for normality using the Shapiro test. The data are presented as means ± S.E. Data were processed by one‐way anova followed by the Tukey–Kramer post hoc test. GraphPad Prism software (version 9, CA, USA) was employed to perform the statistical analysis and establish the represented graphs. The significance level was set to *p* < 0.05 for all statistical tests.^32^

## 3 RESULTS

### 3.1 Thioacetamide‐induced alterations in sera parameters in rats

**FIGURE 1:**
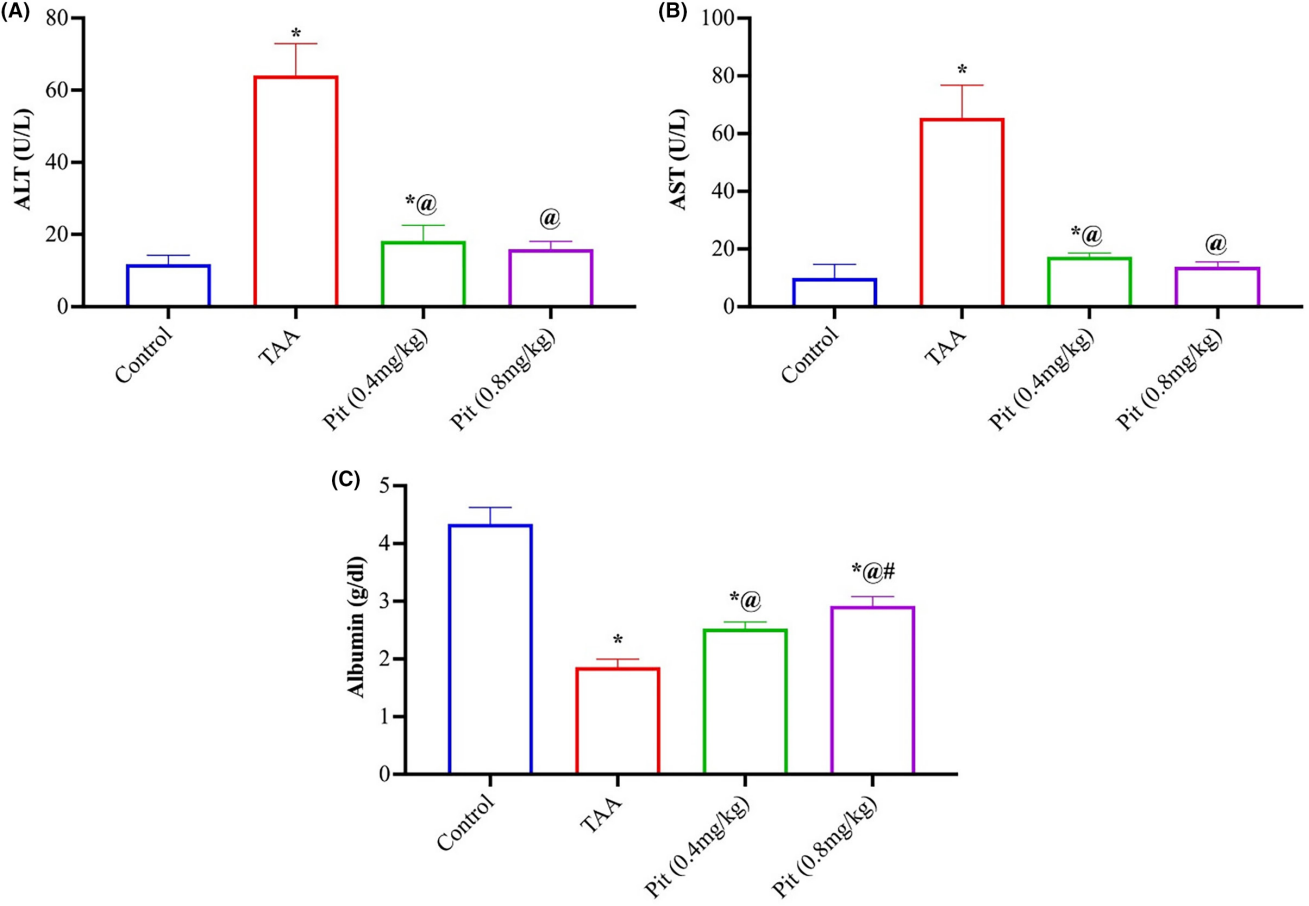
Thioacetamide‐induced alterations in the serum of Wistar rats (A) alanine, (B) aspartate aminotransferases, and (C) albumin. Each bar represents the mean ± SE of six rats. * versus normal control group, ^@^ versus TAA group, ^#^ versus Pit (0.4 mg/kg) at *p* < 0.05. Pit, pitavastatin; TAA, thioacetamide.

Liver damage, as revealed by ALT and AST activity (Figure 1A,B), by 5.4‐ and 6.5‐fold in relation to the negative control, and a reduction in albumin level (Figure 1C) by 57%, as a consequence of TAA exposure (Figure 1C). Pit administration at both 0.4 and 0.8 mg/kg reduced ALT and AST activity by about 75% and elevated the serum albumin level by 36% and 57%, respectively, compared to the TAA group.

### 3.2 Inhibition of the TAA‐Induced oxidative stress by pitavastatin treatment

**FIGURE 2:**
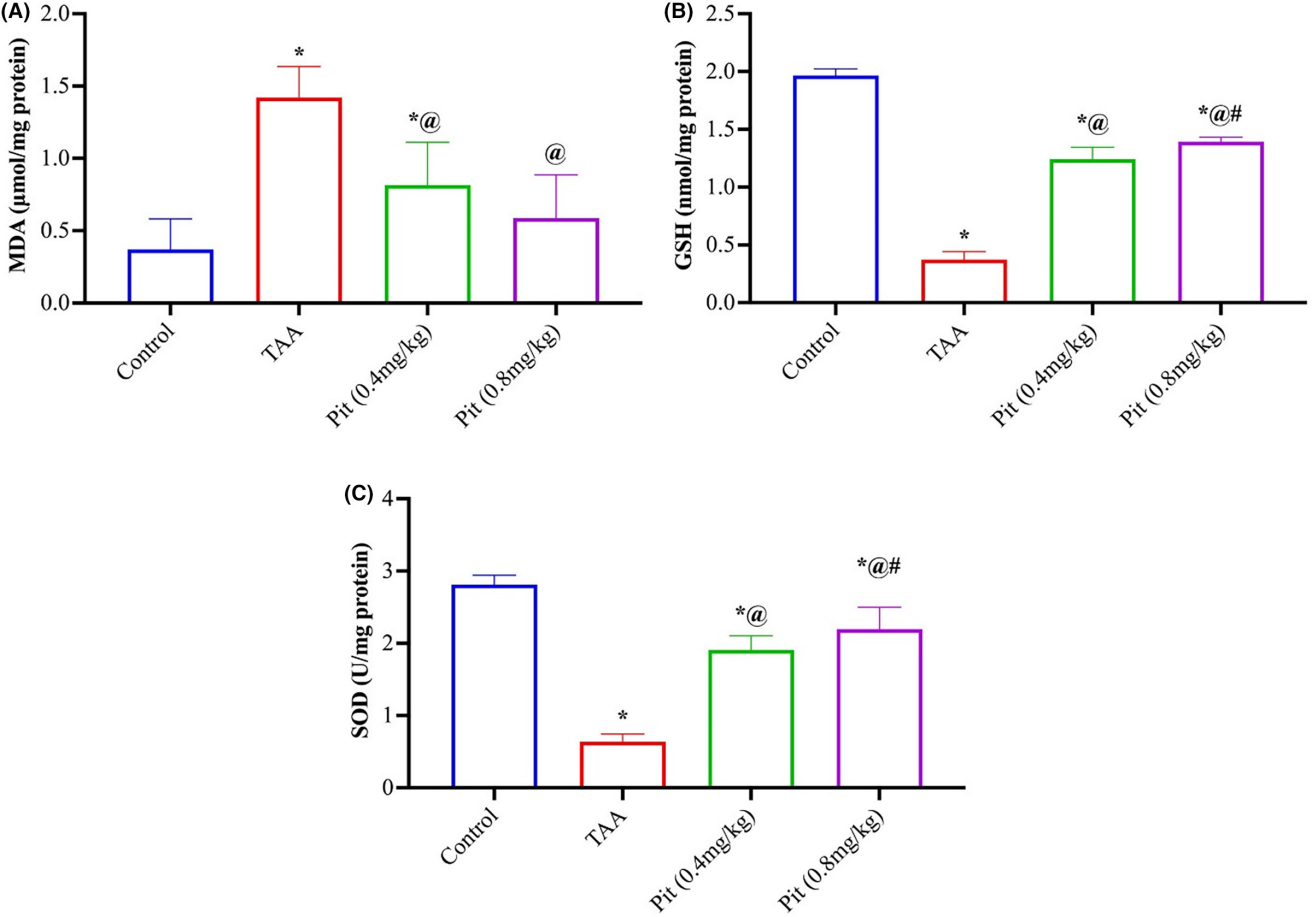
Effect of pitavastatin on (A) malondialdehyde, (B) glutathione, and (C) superoxide dismutase activity in rat livers intoxicated with TAA. Each bar represents the mean ± SE of six rats. * versus normal control group, ^@^ versus TAA group, ^#^ versus Pit (0.4 mg/kg) at *p* < 0.05. Pit, pitavastatin; TAA: thioacetamide.

The degree of lipid peroxidation was evaluated by detecting malondialdehyde (MDA), a result of lipid peroxidation. TAA‐intoxicated rats had a 3.8‐fold rise in MDA levels, while Pit (0.4 or 0.8 mg/kg) treatment suppressed this increase by 43% and 58% relative to the TAA group (Figure 2A). Liver homogenates were tested for GSH level (Figure 2B) and superoxide dismutase (SOD) activity (Figure 2C), an intracellular antioxidant enzyme. TAA caused a decline in both the level of GSH and activity of SOD by 81% and 77% relative to the control group. While the rats given Pit (0.4 or 0.8 mg/kg) recovered the level GSH by 3.3‐ and 3.7‐fold and the activity of SOD by 3.0‐ and 3.4‐fold relative to the TAA group, respectively.

### 3.3 Pitavastatin mitigates TAA‐Induced hepatic inflammation in rats

**FIGURE 3:**
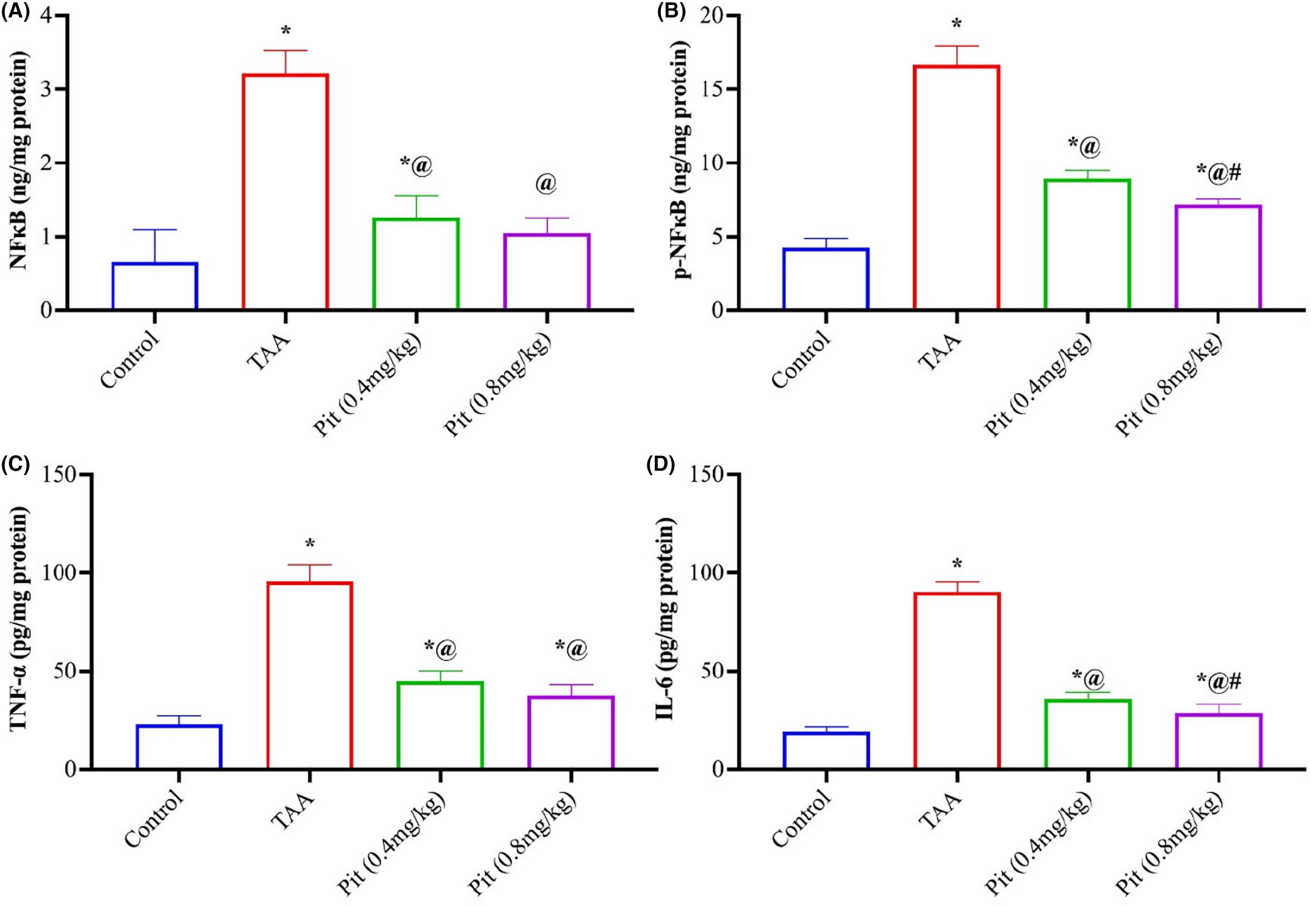
Pitavastatin mitigated inflammation in TAA‐intoxicated rats. Pitavastatin decreased hepatic (A) NF‐κB, (B) p‐NF‐κB, (C) TNF‐α and (D) IL‐6 in TAA rats. Each bar represents the mean ± SE of six rats. * versus normal control group, ^@^ versus TAA group, ^#^ versus Pit (0.4 mg/kg) at *p* < 0.05. Pit, pitavastatin; TAA, thioacetamide.

To further explain the mechanism underlying the protective effect of Pit against liver fibrosis, the levels of NF‐κB, p‐NF‐κB, TNF and IL‐6 were measured to evaluate the anti‐inflammatory activity of Pit. TAA upregulated hepatic NF‐κB (Figure 3A), p‐NF‐κB levels (Figure 3B), TNF‐α (Figure 3C), and IL‐6 (Figure 3D) by 4.8‐, 4‐, 4.1‐ and 4.7‐fold compared to normal control. In contrast, Pit therapy dramatically downregulated NF‐κB or p‐NF‐κB and its downstream signalling proinflammatory mediators, TNF‐α and IL‐6 by 61%, 46%, 53%, and 60% for Pit 0.4 mg/kg dosage and by 68%, 57%, 61%, and 68% for Pit 0.8 mg/kg dose, respectively.

### 3.4 Pitavastatin activates Nrf2/HO‐1 signalling in TAA‐Intoxicated rats

**FIGURE 4:**
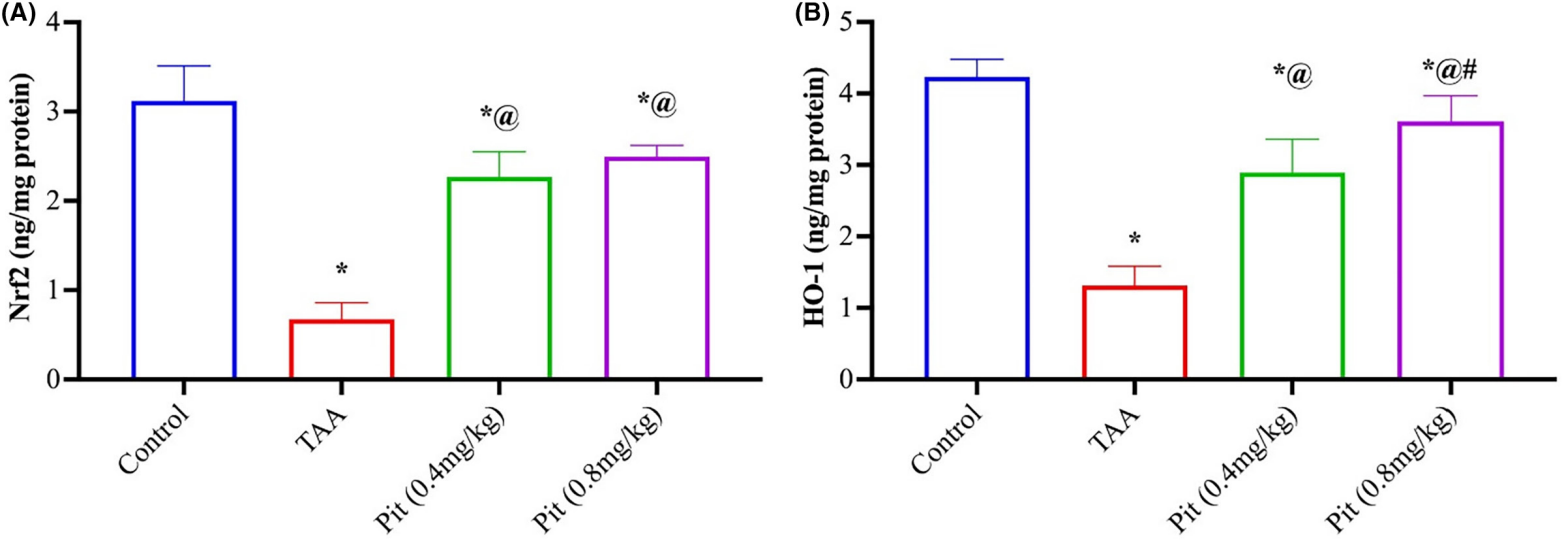
Pit‐activated hepatic (A) Nrf2/ (B) HO‐1 signalling in TAA‐intoxicated rats. Each bar represents the mean ± SE of six rats. * versus normal control group, ^@^ versus TAA group, ^#^ versus Pit (0.4 mg/kg) at *p* < 0.05. Pit, pitavastatin; TAA, thioacetamide.

TAA downregulated the hepatic Nrf2 (Figure 4A) levels compared with the control group by 78%. Pit (0.4 or 0.8 mg/kg) upregulated hepatic Nrf2 by 3.3‐ and 3.7‐fold relative to the TAA group. To confirm the Pit (0.4 or 0.8 mg/kg) activation in TAA‐treated rats, HO‐1, the downstream of Nrf2, was estimated, which showed a prominent rise by 2.2‐ and 2.8‐fold relative to the TAA group (Figure 4B).

### 3.5 Effect of pitavastatin on Nrf2 and PI3K mRNA expression

**FIGURE 5:**
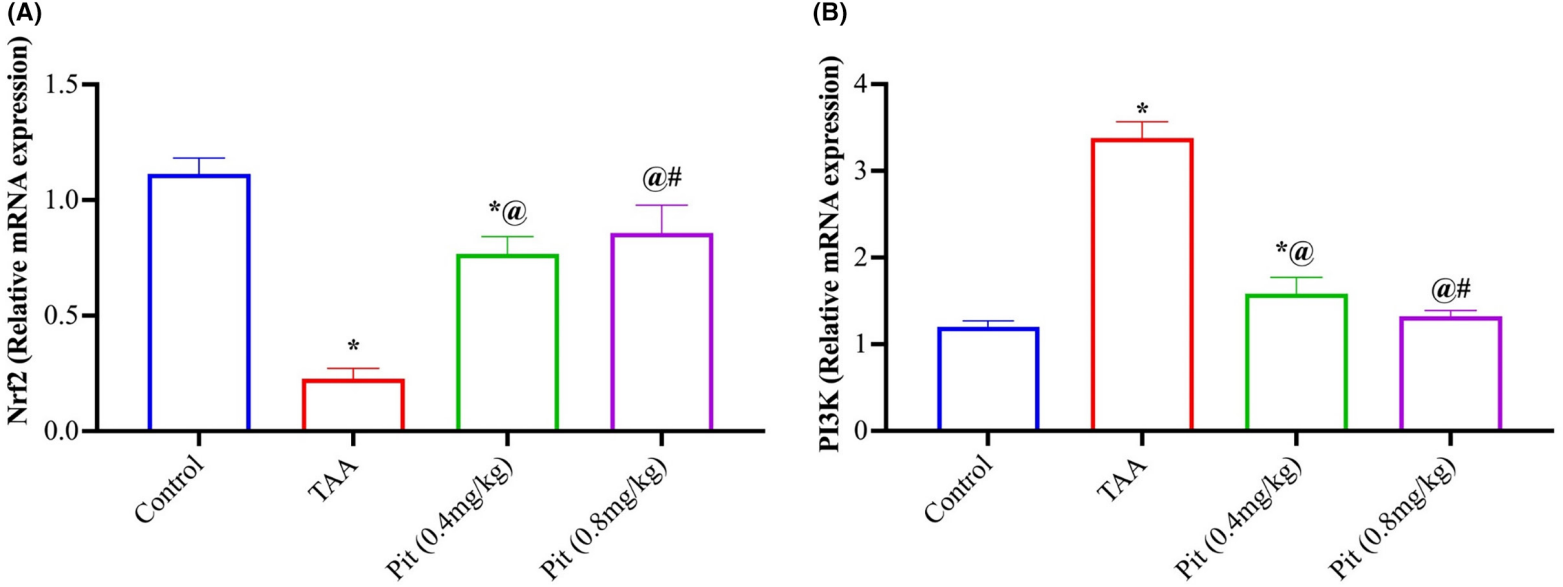
Effect of pitavastatin on Nrf2 and PI3k mRNA expression. Each bar represents the mean ± SE of six rats. * versus normal control group, ^@^ versus TAA group, ^#^ versus Pit (0.4 mg/kg) at *p* < 0.05. Pit, pitavastatin; TAA, thioacetamide.

The mRNA liver content of Nrf2 (Figure 5A) and PI3K (Figure 5B) showed that TAA caused a significant decrease in the Nrf2 and a significant increase in the PI3K by 80% and 2.8‐fold relative to the control group. Pit (0.4 or 0.8 mg/kg) administration upregulated the mRNA of Nrf2 by 3.3‐ and 3.7‐fold and downregulated PI3K by 53% and 61% compared to the TAA group.

### 3.6 Histopathological findings

**FIGURE 6:**
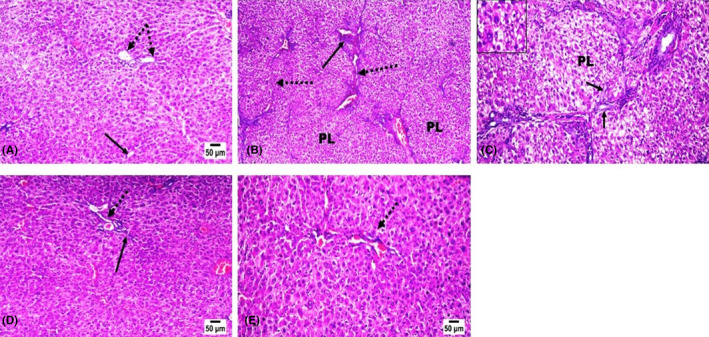
Effect of pitavastatin on histopathology findings. Hematoxylin and eosin stained liver sections. (A) liver of control rat showing normal parenchymal cells, portal triad (dotted arrow), and central vein (arrow). (B, C) liver of TAA administrated rat showing portal to portal bridging fibrosis (dotted arrow) with pseudolobulation (PL) and increased fibrous proliferation in portal areas, few lymphoplasmacytes (long arrow), proliferated bile ducteols (short arrow), vacuolated hepatocytes (insert) and few apoptotic cells. Pit (D) (0.4 mg/kg) and (E) (0.8 mg/kg) treated rats showing marked retraction of fibrous proliferation in portal areas (dotted arrow) and in hepatic parenchyma with only mild incomplete peripheral extension of fibrous septa (arrow) in the low dose treated rat.

Livers of control rats showed normal histological structure, normal central veins, portal areas, and hepatic cords (Figure 6A). Livers of TAA‐cirrhotic model rats revealed capsular corrugation and marked parenchymal fibroplasia with portal‐to‐portal bridging fibrosis (Figure 6B). The portal areas showed increased fibrous tissue proliferation that sends septa extending peripherally, dividing the parenchyma into pseudolobules, some of which are hyperplasic regenerated lobules. Along those sepat, there are proliferated bile ducts, few lymphoplasmacytes, and congested vessels. The hepatic cells within those regenerated nodules are larger in size with large magenta nuclei that sometimes‐compressed other vacuolated hepatic cells (Figure 6C). Regarding rats treated with Pit following TAA administration, examination of their livers showed that Pit could markedly retract hepatic fibroplasia, particularly with the higher dose group. No pseudo‐lobulation was noticed in both groups. With 0.4 mg/kg Pit administration, only mild fibrous proliferation is observed in scattered portal triads with or without incomplete peripheral extension accompanied by mild to moderate degrees of hepatocellular degenerative changes and scattered necrotic cells (Figure 6D). On the other hand, administering Pit at a dose of 0.8 mg/kg markedly curbed fibrous proliferation within the portal areas and hepatic parenchyma. Only some hepatocellular vacuolar degeneration and scarce necrotic cells were the only pronounced lesions (Figure 6E).

### 3.7 Immunohistochemistry findings

**FIGURE 7:**
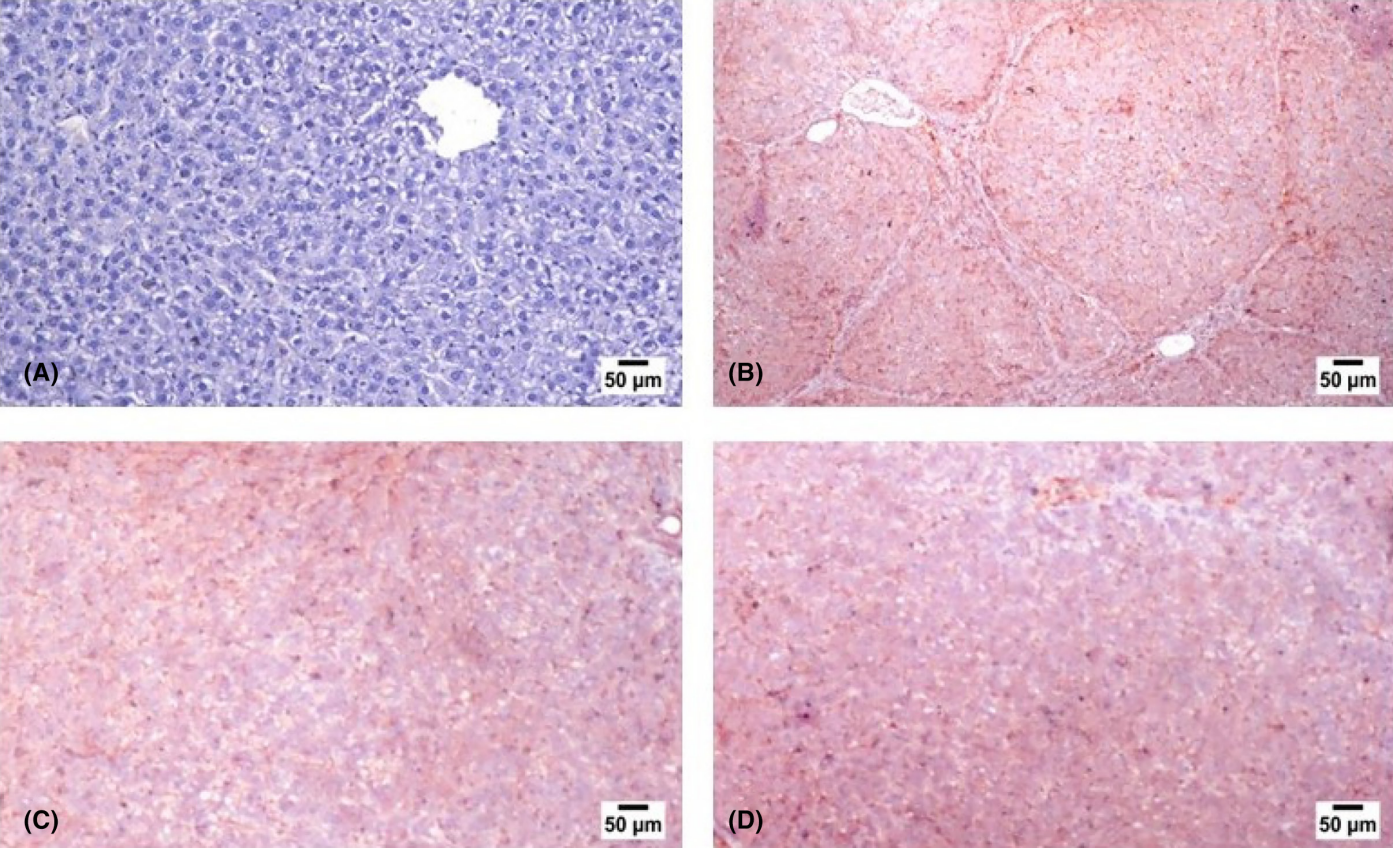
Photograph of liver slices stained with antibodies. Normal control (A), TAA group (B), and Pit‐treated groups 0.4 or 0.8 mg/kg (C,D).

Regarding the *p*‐Akt immune expression, the livers of control rats showed nil expression of *p*‐Akt within the hepatic parenchymal cells (Figure 7A). While in TAA fibrotic model rats, a marked increase in the expression of *p*‐Akt was noticed compared with the other experimental groups (Figure 7B). However, Pit administration at both doses (Figure 7C,D) markedly decreased the immune expression of *p*‐Akt among the livers of the treated rats.

## 4 DISCUSSION

Statins have been recently prescribed for all chronic liver illnesses, and further hepatoprotective benefits are under clinical investigation.^33^ Pit, a third‐generation statin, is of special interest in the treatment of chronic liver disease, including fibrosis, cirrhosis, and hepatocellular carcinoma.^21^, ^26^, ^34^, ^35^ It has the highest bioavailability among all statins (more than 60%); therefore, it is highly effective in low doses and will be readily absorbed with minimal side effects.^36^ The possible hepatoprotective effects of Pit and the underlying mechanisms are being assessed in the current study.

TAA‐induced liver fibrosis is a reliable model that exactly mimics the biochemical and histological changes of human liver fibrosis.^6^ It was employed in the present study as it is associated with lower mortality rates than other models. ALT and AST are cytoplasmic in origin, and elevated serum levels, as shown in the TAA group, reflect cell membrane destruction and hepatocyte death.^32^, ^37^, ^38^, ^39^, ^40^, ^41^ Our results indicated that, unlike all other statins, Pit undergoes minimal hepatic metabolism^42^ and does not cause any elevation in aminotransferase levels. Infect, Pit is a highly lipophilic agent that undergoes glucuronic acid conjugation, and a recent meta‐analysis study demonstrated that only hydrophilic statins result in the risk of aminotransferase elevation.^43^ Therefore, we can state that the high‐dose (0.8 mg/kg) administration of Pit, which showed better control of aminotransferase, was associated with no signs of toxicity in liver fibrosis. In line with our data, the safety of statins for patients with liver dysfunction has also been reported in several clinical trials.^44^

Our results indicate that Pit (0.8 mg/kg) can reverse liver fibrosis by targeting two main pathways—NF‐κB and PI3K/Nrf2/HO‐1—that capture inflammation, oxidative stress, and proliferation. Kupffer cells are an important contributor to HSC/HMF activation and liver fibrosis. They function to sense and remove pathogens and dangerous molecules via pattern‐recognition receptors (PRRs), which detect danger signals, including lipopolysaccharide, chemical insults, and carcinogens.^45^ Upon TAA recognition, various inflammatory cytokines and chemokines were released by Kupfer cells, which stimulated HSCs and started the inflammation response.^6^ TAA also activated NF‐κB, which regulates the inflammatory response in HSCs. This was further confirmed by the high content of Ser536‐phosphorylated p65 (the active form of NF‐κB). Accordingly, several NF‐κB‐dependent genes, including IL‐6 and TNF‐α, were upregulated in the TAA group. Our results demonstrated that Pit exerts a potent anti‐inflammatory effect, most probably via binding to NF‐κB, curbing the formation of its active form, pNF‐κB, and, in turn, inhibiting the downstream inflammatory response of NF‐κB as represented in the reduced liver content of IL‐6 and TNF‐α.^46^

Oxidative stress is a common sequela of inflammation; ROS are highly generated in response to persistent inflammatory mediators. Nrf2 regulates the liver resistance to oxidants^47^; it mediates the expression of antioxidant‐responsive elements (ARE), which in turn, initiate the transcription of several downstream antioxidant protective genes such as HO‐1, SOD, and reduced glutathione (GSH).^48^ Herein, Pit played a dual function in preventing liver fibrosis: first, it greatly enhanced Nrf2 content and expression, allowing Nrf2 to inhibit the activation of hepatic stellate cells. Second, Pit increased the DNA‐binding activity of Nrf2 and induced the expression (measured as liver content) of its target genes, HO‐1, SOD, and GSH. Additionally, Pit—owing to its fundamental lipid‐lowering effect—reduces the levels and oxidation of low‐density lipoprotein; therefore, MDA, a lipid peroxidation byproduct, was significantly decreased upon Pit administration. Collectively, this confirms the cellular protective effect of Pit in liver fibrosis.^49^ It is worth noting that the MDA data presented in Figure 2A exhibited large error bars, particularly in the TAA group. This could be attributed to inherent individual variability in the degree of lipid peroxidation induced by TAA administration. Importantly, despite the variability, pitavastatin treatment at both doses still significantly reduced MDA levels compared to the TAA group.^35^, ^37^, ^50^, ^51^ The lack of dose‐dependent difference in MDA suppression between the two pitavastatin doses could suggest a potential ceiling effect on lipid peroxidation inhibition even at the lower 0.4 mg/kg dose. However, further studies with larger sample sizes may be warranted to fully evaluate the dose–response relationship.

The PI3K/AKT signalling pathway is intimately connected to the activation of hepatic stellate cells and the production of ECM.^52^, ^53^ Inhibition of the PI3K/AKT signalling has been shown to be effective in preventing liver damage, enhancing liver function, and reducing collagen synthesis and deposition.^54^, ^55^ Therefore, one of the current approaches for treating liver fibrosis is to reduce PI3K/AKT activity. According to our findings, TAA increased the expression of PI3K and *p*‐AKT, which is consistent with other research.^56^, ^57^ In line with our findings in the rat model of TAA‐induced hepatic fibrosis, the PCR and immunohistochemical analysis showed that Pit efficiently suppresses the PI3K/AKT signal pathway.^58^ These findings showed that the suppression of the PI3K/AKT signalling pathway by Pit could reduce hepatic fibrosis. Additionally, NF‐κB signalling is governed by PI3K/AKT.^59^ Crosstalk between the PI3K/AKT and NF‐κB pathways may have occurred in the current investigation because both of their activity was suppressed.

Beyond its lipid‐lowering role, our study showed that Pit has beneficial pleiotropic effects that target key processes in the pathophysiology of liver fibrosis. It acts on inflammation by decreasing the production of NF‐kB, and hence the release of proinflammatory cytokines such as TNF‐α and IL‐6, and it decreases the level of oxidative stress by stimulating the Nrf2/HO‐1 pathways.

It is important to note that our investigation focused on a concise acute model, facilitating the monitoring of liver fibrosis progression. This approach serves as a valuable clinical tool for safeguarding patients' livers, preventing the progression to advanced stages of hepatic fibrosis. Moreover, it provided a reliable means to histologically induce substantial bridging fibrosis while mitigating the risk of increased animal mortality associated with more prolonged exposures. This model was studied previously in studies.^27^, ^37^, ^50^, ^51^, ^60^

The observed antifibrotic effects and mechanisms of pitavastatin in a rat model of TAA‐induced liver fibrosis suggest its potential for clinical use. Given its existing approval and favourable safety profile, repurposing pitavastatin for hepatic fibrosis treatment in patients appears feasible and could offer a streamlined path for clinical translation. However, additional pharmacokinetic studies are required to determine the optimal dosing regimen that can safely replicate the antifibrotic concentrations achieved in this preclinical study. The next steps should involve clinical trials assessing pitavastatin's efficacy as a monotherapy or in combination with other agents for liver fibrosis across various causes. In summary, this study establishes a robust preclinical foundation, supporting the need for future clinical investigations into pitavastatin as a therapy for liver fibrosis.

## 5 CONCLUSIONS

The current investigation results show that Pit is very efficient in reducing TAA‐induced liver fibrosis, which is probably mediated by its ability to inhibit oxidative stress and lipid peroxidation as an antioxidant. It has the ability to reduce inflammation by blocking the NF‐κB pathway, which triggers the production of inflammatory mediators like TNF‐α and IL‐6. As a result of activating Nrf2, increasing HO‐1, suppressing PI3K activity, and inhibiting Akt phosphorylation, Pit therapy improved the oxidative stress state of rat livers. This indicates that this improvement is associated with the Nrf2/HO‐1 signalling pathway. Pit exhibited antifibrotic properties against TAA‐induced liver fibrosis in rats. These findings suggest that Pit may have therapeutic potential for reducing hepatic inflammation and its development into fibrosis.

The antifibrotic effects and mechanisms demonstrated for pitavastatin in the rat model of TAA‐induced liver fibrosis provide support for its potential clinical application. As pitavastatin is already an approved medication with a favourable safety profile, repurposing it for hepatic fibrosis treatment in patients is feasible and could provide an expedited path for clinical translation. Further pharmacokinetic studies are still needed to determine the optimal dosing regimen that could safely reproduce the antifibrotic hepatic concentrations achieved in this preclinical study. Clinical trials evaluating the antifibrotic efficacy of pitavastatin monotherapy or in combination with other agents in patients with liver fibrosis of various aetiologies are warranted. Overall, the present study provides a strong preclinical basis to motivate future clinical investigation of pitavastatin for liver fibrosis therapy.

## AUTHOR CONTRIBUTIONS

**Marawan Elbaset:** Conceptualization (equal); formal analysis (equal); investigation (equal); methodology (equal); project administration (equal); software (equal); supervision (equal); validation (equal); visualization (equal); writing – original draft (equal); writing – review and editing (equal). **Bassim Mohamed:** Project administration (equal); resources (equal); supervision (equal); visualization (equal); writing – original draft (equal); writing – review and editing (equal). **Alyaa Hessin:** Resources (equal); writing – original draft (equal). **Sahar Abd El‐Rahman:** Investigation (equal); resources (equal); software (equal). **Tuba Esatbeyoglu:** Funding acquisition (equal); project administration (equal); resources (equal); writing – review and editing (lead). **Sherif Afifi:** Resources (equal); writing – review and editing (equal). **Hany Fayed:** Conceptualization (equal); formal analysis (equal); investigation (equal); methodology (equal); project administration (equal); supervision (equal); visualization (equal); writing – original draft (equal); writing – review and editing (equal).

## FUNDING INFORMATION

The publication of this article was funded by the Open Access Fund of Leibniz Universität Hannover.

## CONFLICT OF INTEREST STATEMENT

The authors confirm that there are no conflicts of interest.

## Data Availability

The data that support the findings of this study are available from the corresponding author upon reasonable request.
